# Application of a Bayesian non-linear model hybrid scheme to sequence data for genomic prediction and QTL mapping

**DOI:** 10.1186/s12864-017-4030-x

**Published:** 2017-08-15

**Authors:** Tingting Wang, Yi-Ping Phoebe Chen, Iona M. MacLeod, Jennie E. Pryce, Michael E. Goddard, Ben J. Hayes

**Affiliations:** 1 School of Engineering and Mathematical Sciences, La Trobe University, Melbourne, VIC 3083 Australia; 2 Agriculture Victoria, AgriBio, Centre for AgriBioscience, Melbourne, VIC 3083 Australia; 3 Dairy Futures Cooperative Research Centre, Melbourne, VIC 3083 Australia; 4 School of Applied Systems Biology, La Trobe University, Melbourne, VIC 3083 Australia; 5 Faculty of Veterinary and Agricultural Science, University of Melbourne, Melbourne, VIC 3010 Australia; 6 Queensland Alliance for Agriculture and Food Innovation, Centre for Animal Science, University of Queensland, St Lucia, Brisbane, QLD 4072 Australia

## Abstract

**Background:**

Using whole genome sequence data might improve genomic prediction accuracy, when compared with high-density SNP arrays, and could lead to identification of casual mutations affecting complex traits. For some traits, the most accurate genomic predictions are achieved with non-linear Bayesian methods. However, as the number of variants and the size of the reference population increase, the computational time required to implement these Bayesian methods (typically with Monte Carlo Markov Chain sampling) becomes unfeasibly long.

**Results:**

Here, we applied a new method, HyB_BR (for Hybrid BayesR), which implements a mixture model of normal distributions and hybridizes an Expectation-Maximization (EM) algorithm followed by Markov Chain Monte Carlo (MCMC) sampling, to genomic prediction in a large dairy cattle population with imputed whole genome sequence data. The imputed whole genome sequence data included 994,019 variant genotypes of 16,214 Holstein and Jersey bulls and cows. Traits included fat yield, milk volume, protein kg, fat% and protein% in milk, as well as fertility and heat tolerance. HyB_BR achieved genomic prediction accuracies as high as the full MCMC implementation of BayesR, both for predicting a validation set of Holstein and Jersey bulls (multi-breed prediction) and a validation set of Australian Red bulls (across-breed prediction). HyB_BR had a ten fold reduction in compute time, compared with the MCMC implementation of BayesR (48 hours versus 594 hours). We also demonstrate that in many cases HyB_BR identified sequence variants with a high posterior probability of affecting the milk production or fertility traits that were similar to those identified in BayesR. For heat tolerance, both HyB_BR and BayesR found variants in or close to promising candidate genes associated with this trait and not detected by previous studies.

**Conclusions:**

The results demonstrate that HyB_BR is a feasible method for simultaneous genomic prediction and QTL mapping with whole genome sequence in large reference populations.

## Background

Whole genome sequence data is available for an increasing number of species. In some cases enough individuals have been sequenced to serve as a reference panel for imputation of individuals that have been genotyped with SNP arrays to whole genome sequence variant genotypes. A good example of such a reference set is the 1000 bull genomes project which includes 234 bulls with whole-genome sequencing data and 28.3 million genotyped sequence variants [1]. Compared with dense SNP arrays, the advantage of using whole genome sequence data might potentially include more accurate genomic predictions within and across breeds [2–5], better persistence of accuracy of genomic predictions across generations, and more precise QTL mapping [5], all as a result of including the causal mutation genotypes in the data set.

As the resulting data sets will be extremely large (thousands of individuals with millions of imputed genotypes), the algorithms used to derive genomic predictions must be computationally efficient. Ideally, they should also implement a non-linear model at the level of the SNP effects, including the possibility of excluding some SNPs from the model, as such models have been demonstrated to give higher accuracies of genomic predictions for some traits with high-density genotype data [5, 6]. Although computationally efficient, GBLUP and BLUP do not satisfy the second criteria (they implement a linear model and all SNPs are always in the genomic prediction model). BayesR [7] is a flexible non-linear model, which assumes that SNP effects follow a mixture of four normal distributions (with zero variance, very small variance, small variance, and moderate variance). Compared with GBLUP, BayesR results in superior accuracy of genomic prediction for some traits [6, 8–12]. However, as Bayesian models are typically implemented with MCMC (Markov Chain Monte Carlo) sampling, application of BayesR with sequence data is currently not feasible.

Another advantage of non-linear models such as BayesR, is the application of QTL mapping [5, 6, 8, 13, 14]. Loh et al. [14] pointed out that Bayesian mixed-models with speed-up schemes (termed fastBayesB [15]) could improve the power of detecting genes associated with human diseases. There are several modified versions of Bayesian model implemented for the identification of causal mutations. Speed and Balding [13] developed an efficient approach termed multiBLUP (a mixture model of SNP effects, similar to nonlinear models), which was applied on the Welcome Trust Case Control Consortium (WTCCC) human disease data. Later, Kemper et al. [6] implemented a nonlinear model (BayesR) for mapping QTL to 250 kb windows in dairy cattle. Then, Moser et al. [8] applied a modified version of BayesR (updating the additive genetic variance in the MCMC chain instead of fixing it, as in the original BayesR) to WTCCC human disease data. Furthermore, MacLeod et al. [5] proposed the algorithm referred to as BayesRC, which is a modified version of BayesR incorporating biological prior information. All these studies have demonstrated that nonlinear models, which might exclude SNPs from the models with the assumptions of Bayesian mixture priors for SNP effects, could actually help to improve the precision of QTL mapping or association studies in human or dairy cattle.

To take advantage of the accuracy superiority of MCMC nonlinear models but improve their time-efficiency, a hybrid scheme (termed HyB_BR) was proposed by Wang et al. [16]. This scheme has three steps: 1) Implement the mixture model of BayesR, which had been demonstrated to be quite flexible for genomic prediction; 2) run an expectation-maximisation algorithm that estimates the parameters in the mixture model; 3) Using the solutions from the EM as starting points, run a limited number of MCMC iterations to improve the parameter estimates. The results of the Hybrid algorithm on 600 K SNP data in dairy cattle data and 300 K SNP data in human disease data from Welcome Trust Case Control Consortium (WTCCC) have demonstrated that the Hybrid algorithm performed as well as BayesR while requiring half of the running time demanded by MCMC iterations [16].

With the aim of investigating whether HyB_BR gave comparable accuracies to BayesR with MCMC for genomic prediction and precision of QTL mapping with whole genome sequence data, we implemented HyB_BR on a large subset of imputed whole-genome sequence data with 994,019 variants in 16,214 cattle. The genotype data came from the imputed sequence variants in or close to gene coding regions and some SNP from the 600 K Bovine HD SNP genotypes. The HyB_BR algorithm was evaluated on this data set with three criteria: 1) computational performance (speed) compared to a full MCMC implementation, 2) prediction accuracy for a range of complex traits with different genetic architecture. The traits included fat yield, milk yield, protein yield, fat percent, protein percent, fertility and heat tolerance and 3) the precision of HyB_BR for QTL mapping of milk production traits, fertility and heat tolerance.

## Methods

### High density and sequence genotypes

Two types of genomic data, 600 K Bovine HD SNP array, and imputed sequence data were used in this study. As described by Kemper et al. (2015) [6], 10,311 Holstein, 4738 Jersey and 249 Australian Red bulls and cows were genotyped with the Bovine SNP50 Array (Illumina, San Diego, CA). In addition, 1620 Holstein bulls and cows, 125 Jersey bulls, and 114 Australian Red bulls were genotyped with the 777 K bovine HD SNP panel. After quality control steps described by Erbe et al. (2012) [7], all genotypes were imputed to 632,003 SNP using Beagle 3.0 [17].

For the Sequence data set (termed SEQ), the sequences of 136 Holstein and 27 Jersey bulls from the 1000 Bulls Genome Project [1] were used as a reference set for imputation. All the animals described above with real or imputed 600 K SNP genotypes were imputed to whole genome sequence data using Beagle 3.0 software [18]. In total there were 2.785 million sequence variants imputed, including both SNPs and indels in either coding regions or putative regulatory regions flanking genes [5]. After quality control including minor allele frequency filtering and LD pruning by PLINK [19], there were 994,019 variants remaining including 370,259 markers from the 600 K SNP panel, and 623,760 sequence variants in gene coding regions or 5000 bp up- and down-stream of the gene start stop positions as detailed by MacLeod et al. (2016) [5].

### Phenotypes

Protein, fat and milk yields and, fat and protein percent are key traits in dairy cattle breeding. Phenotypes that were pre-corrected for fixed effects (herd-year-season, and lactation number) were used in this study, these are known as trait deviations (TDs) and daughter trait deviations (DTDs) for cows and bulls respectively. TDs and DTDs are provided by DataGene (and its predecessor, the Australian Dairy Herd Improvement Scheme), which is the organisation responsible for providing genetic evaluations to the Australian dairy industry. (e.g. DataGene; http://datagene.com.au/index.php). A summary of the phenotypes is shown in Table 1. For milk production traits, there were 16,214 bulls and cows from Holstein and Jersey breeds as the reference set. Then, for the validation sets, Holstein and Jersey bulls were used to assess the accuracy of within-breed prediction. These bulls were the youngest cohorts (born after 2005) in the data set. As mentioned in [6], all the bulls of the validation set have more than 20 effective daughters. In addition, Australian Red bulls (a third breed; not included in the reference set) were included for the validation set to evaluate the performance of across-breed prediction. We implemented the calculation of Garrick et al. (2009) [20] to appropriately weight the phenotypes of bulls and cows as follows:

$$ {w}_i(bulls)=\frac{\left(1-{h}^2\right)}{ch^2+\left(4-{h}^2\right)/d},\mathrm{and}\ {w}_i(cows)=\frac{\left(1-{h}^2\right)}{ch^2+\left[1+\left(r-1\right)t\right]/r-{h}^2}, $$

**Table 1:** The number of animals in the reference sets and validation sets

Traits	Reference sets				Validation sets		
	Holstein		Jersey		Holstein Bulls	Jersey Bulls	Australian Red Bulls
	Bulls	Cows	Bulls	Cows			
Milk production traits (FatY/MilkY/ProtY/Fat%/Protein%)	3049	8478	770	3917	262	105	114
Fertility	2806	7838	716	3830	396	81	114
Heat Tolerance traits (FatY_HT/MilkY_HT /ProtY_HT)	2028	2037	476	1116	252	101	-

Milk production traits include fat yield (*FatY*), milk yield (*MilkY*), protein yield (*ProteinY*), fat percent (*Fat%*) and protein percent (*Protein%*); Heat tolerance traits are the decline of fat yield (*FatY_HT*), milk yield (*MilkY_HT*) and protein yield (*ProtY_HT*) under heat stress

where, *h* ^2^ is the heritability of the trait; *t* is the repeatability of the traits; *d* is the number of the daughter of each bulls; *r* is the number of records; *c* is the proportion of additive genetic variance not accounted for by the SNP [20]. To compare the prediction accuracy of GBLUP, BayesR and HyB_BR for multi-breeds and across-breed, the weight calculation is included in all three models.

In addition to milk production traits, fertility is another important complex trait. The DTD and TD that DataGene calculate and that was available to this study was calving interval (CI) which is the number of days between consecutive calving, For fertility, the number of bulls and cows in the reference set, i.e. with genotypes and fertility phenotypes was around 15,190. The validation set includes Holstein bulls (youngest cohort born after 2004) and Jersey bulls (youngest cohort born after 2005).

As weather becomes warmer and less predictable, there is growing interest in developing genomic breeding values for heat tolerance [21]. In Australian dairy genetics studies, heat tolerance is defined as the rate of the decline in production traits (e.g. fat, milk and protein yield) with increasing heat stress [21]. The rate of decline for each trait was estimated for each cow in the data set with a linear random regression of yield on daily temperature-humidity index (THI), when THI was above a threshold of 60 units [21–23]. The total number of animals with phenotypes for heat tolerance was 5657 and included Holstein and Jersey cows and bulls. The validation set for heat tolerance was a set of Holstein bulls and a set of Jersey bulls, Table 1. In contrast to the milk production and fertility phenotypes, heat tolerance is still under development and is not yet officially released as a breeding value in Australia.

The input parameters for HyB_BR were estimated from the data with ASReml4 [24] and included additive genetic variance, error variance, and additive polygenic variance (Table 2). Using the variances, the heritability is calculated based on the “narrow-sense” definition [25] as the ratio of additive genetic variance and the sum of additive genetic variance, error variance and additive polygenetic variance (${\boldsymbol{h}}^2={\boldsymbol{\sigma}}_{\boldsymbol{g}}^2/\left({\boldsymbol{\sigma}}_{\boldsymbol{g}}^2+{\boldsymbol{\sigma}}_{\boldsymbol{a}}^2+{\boldsymbol{\upsigma}}_{\boldsymbol{e}}^2\right)$). The heritability for milk production traits is consistent with the published results of Kemper et al. (2015) [6]. Compared with milk production traits, heritabilities for heat tolerance traits and fertility were lower. Across all the traits, the prediction accuracy is evaluated using the correlations between genomic estimated breeding value (GEBV) and DTD in the validation sets. The regression of DTD on GEBV in the validation sets was used to investigate if any of the methods resulted in biased predictions.

**Table 2:** The genetic architecture of milk production traits and Fertility estimated by ASReml

	Additive genetic variance (${\sigma}_g^2$)	Additive polygenic variance (${\sigma}_a^2$)	Error variance (${\upsigma}_e^2$)	Heritability (*h* ^2^)
FatY	118.594	48.689	234.326	0.421
MilkY	114.827e + 03	38.532e + 03	135.598e + 03	0.528
ProtY	72.488	36.072	140.417	0.443
Fat%	0.056	0.008	0.018	0.781
Protein%	0.012	0.003	0.003	0.818
Fertility	42.990	0.003e-01	340.287e-01	0.013
FatY_HT^a^	0.041	0.581e-07	0.571	0.072
MilkY_HT^a^	0.004	0.353e-06	0.035	0.091
ProtY_HT^a^	0.035	0.564e-07	0.561	0.059

^a^Labels the traits of Fat yield, milk yield, and protein yield under heat stress

### Genomic prediction methods

#### GBLUP

GBLUP assumes all marker effects follow a normal distribution with the same additive genetic variance. The overall model of GBLUP is:

1 $$ \mathbf{y}=\mathbf{X}\boldsymbol{\upbeta } +\mathbf{Su}+\mathbf{Wv}+\mathbf{e} $$

Where,

**y **= vector of *n* phenotypes.

**β **= vector of *b* fixed effects, following uninformative priors.

**u **= vector of *q* random genetic effects (*q* = number of animals) captured by the SNP, with $N\left(0,\mathbf{G}{\sigma}_g^2\right)$. **G** is the *q* x *q* genomic similarity matrix between pairs of individuals constructed as described by [26]; ${\sigma}_g^2$ is the additive genetic variance.

**v **= vector of *q* additive polygenic effects (*q* = number of animals), with $\mathbf{v}\sim N\Big(0,\mathbf{A}{\sigma}_a^2$). **A** is the *q* × *q* pedigree-based relationship matrix, and ${\sigma}_a^2$ is the additive polygenic variance.

**e**= vector of *n* residual errors. For cattle data, $\mathbf{e}\sim N\left(0,\mathbf{E}{\upsigma}_e^2\right)$, the *n* × *n* diagonal matrix **E** is especially designed to evaluate the different contributions of the phenotype records from different sex to the error variance, de-regressing predicted breeding values and weighting information for genomic regression analyses [20].

**X** = *n* × *b* design matrix, allocating phenotypes **y** to fixed effects **β**. *b* is the number of fixed effects

**W**= *n* × *q* design matrix, which aims at allocating the *q* × 1 vector of polygenic effects to **y**.

**S** = *n* × *q* design matrix, allocating the *q* × 1 vector of genetic values to **y**.

#### BayesR

Compared with the common prior distributions of GBLUP, BayesR [7] assumes SNP effects are drawn from the mixture of four normal distributions. BayesR aims at estimating each SNP effects instead of estimating breeding values directly for each animal. Therefore, the genetic value **u** in the model (1) is substituted with **Zg** in the BayesR model. Briefly, the data model of BayesR can be written as:

2 $$ \mathbf{y}=\mathbf{X}\boldsymbol{\upbeta } +\mathbf{Zg}+\mathbf{Wv}+\mathbf{e} $$

Where,

**g** = *m* vector of SNP effects, $\mathbf{g}\sim N\left(0,\mathrm{I}{\boldsymbol{\upsigma}}_{\mathbf{i}}^2\right)$, ${\boldsymbol{\upsigma}}_{\mathbf{i}}^2=\left\{0,{0.0001}^{\ast }{\upsigma}_{\mathrm{g}}^2,{0.001}^{\ast }{\upsigma}_{\mathrm{g}}^2,{0.01}^{\ast }{\upsigma}_{\mathrm{g}}^2\right\}$. Therefore, each SNP have four possible normal distributions: $N\Big(0,{0}^{\ast }{\sigma}_g^2$), $N\Big(0,{0.0001}^{\ast }{\sigma}_g^2$), $N\Big(0,{0.001}^{\ast }{\sigma}_g^2$), and $N\Big(0,{0.01}^{\ast }{\sigma}_g^2$). Related to such mixture priors, there are two other parameters including *b*(*i*, *k*) and Pr.

*b*(*i*, *k*) = {0, 1}, which defines whether or nor SNP *i* follows normal distribution *k* (*k* = 1 , 2 , 3 , 4). Therefore, the prior distribution of each SNP *i* conditional on *b*(*i*, *k*) can be written as:

$$ \mathrm{p}\left(\left.{g}_i\right|b\left(i,k\right)\right)=\left\{\begin{array}{cc}\hfill 0,\hfill & \hfill b\left(i,k\right)=1\hfill \\ {}\hfill \frac{1}{\sqrt{2\pi {\upsigma}_{\mathrm{i}}^2\left[k\right]}}\exp \left(-\frac{g_i^2}{2{\upsigma}_{\mathrm{i}}^2\left[k\right]}\right),\hfill & \hfill b\left(i,k\right)=1\left(k=2,3,4\right)\hfill \end{array}\right.. $$

Pr= the vector of proportion parameter, which defines the proportion SNPs in each of four normal distributions. The prior of Pr is drawn from Dirichlet distribution Pr ~ Dirichlet(α), with **α** = [1, 1, 1, 1]. The conditional distribution of SNP effect on the proportion parameter Pr is:

$$ p\left({g}_i|\mathbf{\Pr}\right)={Pr}_1\times N\left(0,{0}^{\ast }{\sigma}_g^2\right)+P{r}_2\times N\left(0,{0.0001}^{\ast }{\sigma}_g^2\right)+{Pr}_3\times N\left(0,{0.001}^{\ast }{\sigma}_g^2\right)+{Pr}_4\times N\left(0,{0.01}^{\ast }{\sigma}_g^2\right). $$

**Z** is the standardised (for mean and variance) *n* × *m* genotype matrix.

To implement the BayesR model, and arrive at posterior estimates of parameters, Gibbs sampling has been used, as described by Kemper et al. (2015) [6]. On the sequence data, we use five independent replicate chains of the Gibbs sampling, and for each independent chain, there are 40,000 iterations, with the first 20,000 iterations discarded as burn in, as described by Kemper et al. (2015) (for 630 K SNP data).

#### HyB_BR

The HyB_BR model [16] incorporates the same assumption for SNP effects as BayesR, but serially hybridizes the expectation-maximization (EM) and MCMC to reduce large number of iterations required by MCMC. That is, HyB_BR first implements an EM algorithm to perform the Maximum A Posterior (MAP) estimation until converged. Then, to improve accuracy, a limited number of MCMC iterations are performed to improve parameter estimates [16].

As described in Wang et al. (2016) [16], the HyB_BR model for a SNP effect is:

3 $$ \mathbf{y}=\mathbf{X}\boldsymbol{\upbeta } +{\mathbf{Z}}_{\boldsymbol{i}}{\mathrm{g}}_{\mathrm{i}}+\mathbf{u}+\mathbf{Wv}+\mathbf{e} $$

**Fig. 1:**
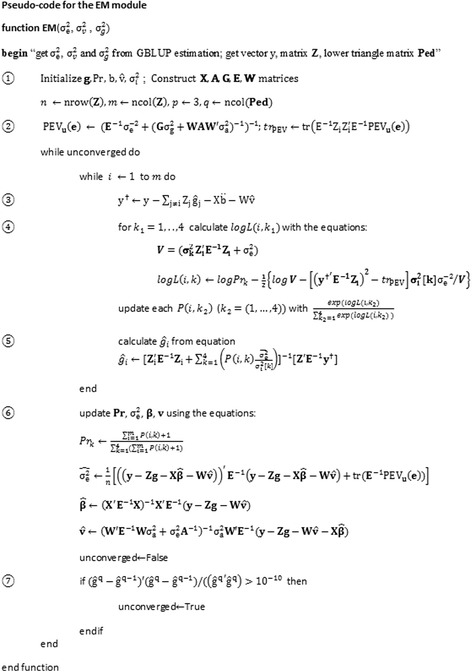
The pseudo-code of the EM module

Assumptions in the model are 1) each SNP effect g_i_ follows the same prior assumption as BayesR with **Z**_***i***_ being the standardized genotype for SNP *i*. 2) to correct the prediction errors generated by all other SNPs, HyB_BR introduces the genetic values **u**, whereby a correction based on the prediction error variance (PEV) is introduced to account for the effects of all the other SNP with a GBLUP model as detailed by Wang et al. [16]. Then under the model (3), the posterior distribution for all related parameter sets {*g*_*i*_ , Pr  , **β** , **u** , **v**, ${\upsigma}_e^2$} are derived according to the theory: *p*(*θ*| **y**) ∝ *f*(**y**| *θ*)*p*(*θ*), where *f*(**y**| *θ*) is the likelihood function based on model (3) and *p*(*θ*) is the prior density function for the parameter sets *θ*. Based on the derived marginal posterior distribution *p*(*θ*| **y**), the expectation- maximization steps are implemented to estimate each parameter while “integrating out” the other parameters detailed by Wang et al. (2016). The process of the EM module is presented in pseudo code in Fig. 1.

As shown in Fig. 1, the EM module begins by initializing all the input parameters including SNP effects (**g**), Proportion parameter (Pr), the variance for each SNP ($$ {\boldsymbol{\upsigma}}_{\mathbf{i}}^2 $$), the fixed matrix (**X**), the pedigree based relationship matrix (**A**), the genomic relationship matrix (**G**), the error matrix (**E**), and index matrix for polygenic effects (**W**). Similar to emBayesR [27], the starting values of ***g*** and Pr are set as ***g*** = 0.01 and Pr = {0.5, 0.487, 0.01, 0.003}, while $$ {\boldsymbol{\upsigma}}_{\mathbf{i}}^2=\left\{0,{0.0001}^{\ast }{\upsigma}_{\mathrm{g}}^2,{0.001}^{\ast }{\upsigma}_{\mathrm{g}}^2,{0.01}^{\ast }{\upsigma}_{\mathrm{g}}^2\right\} $$. The additive genetic variance $$ {\upsigma}_{\mathrm{g}}^2 $$, error variance $$ {\upsigma}_{\mathrm{e}}^2 $$, and polygenic variance $$ {\upsigma}_{\mathrm{a}}^2 $$ are obtained from ASReml. Later, HyB_BR fixes the value of the additive genetic variance and additive polygenic variance (not updating them in later MCMC and EM iterations). The *n*×3 matrix **X** is a design matrix, allocating the phenotypes to fixed effects. In our case, matrix **X** is set up with first column being the mean, the second and third columns defining the breeds (Holstein and Jersey) and sex (bulls and cows) of the cattle. The pedigree relationship matrix, **A,** is built using the lower symmetrical matrix **Ped** detailed by Henderson [28]; while the genomic relationship matrix **G** is constructed using the equation **G** = **Z** ^*s*^ **Z** ^*s*′^/*n*, **Z** ^*s*^ is the standardized **Z** matrix with $$ {{\mathbf{Z}}^s}_{ij}=\raisebox{1ex}{$\left({\mathbf{Z}}_{ij}-2{p}_i\right)$}\!\left/ \!\raisebox{-1ex}{$\sqrt{2{p}_i\left(1-{p}_i\right)}$}\right. $$. The diagonal error matrix **E** is constructed according to the equation defined by Garrick et al. [20] and described above for the phenotypes used in this study.

The EM steps require the time complexity *O*(*mn*). For the calculation of $tr\left({\mathbf{E}}^{-1}{\mathbf{Z}}_{\mathbf{i}}{\mathbf{Z}}_{\mathbf{i}}^{\prime }{\mathbf{E}}^{-1}{\mathrm{PEV}}_{\mathbf{u}}\left(\mathbf{e}\right)\right)$ which is calculated prior to the EM steps, the required time is *O*(*m* ^2^ *n*). This calculation accounts for 40% of the total computational time of EM module. Since the calculation is independent for each SNP, we parallelize the operations by chromosomes, which reduce total running time by approximately 30%.

Once the EM has converged using the criterion ($$ {\left({\widehat{\mathrm{g}}}^{\mathrm{q}}-{\widehat{\mathrm{g}}^{\mathrm{q}-1}}\right)^{\prime}}\left({\widehat{\mathrm{g}}}^{\mathrm{q}}
-{\widehat{\mathrm{g}}^{\mathrm{q}-1}}\right)/\Big(\left({\widehat{\mathrm{g}}}
^{\mathrm{q}^{\prime}}\,\,{\widehat{\mathrm{g}}}^{\mathrm{q}}\right)\,.<{10}^{-10}$$ with *q* the iteration number, the parameter estimates from the EM are used as starting points of parameter values in the MCMC iterations. The steps of MCMC iterations iteration were detailed by Kemper et al. (2015) [6]. Furthermore, Wang et al. (2016) [16] suggested a speed-up scheme to improve computational efficiency. The scheme is as follows. After 500 MCMC iterations, the SNPs with high probability in the distribution with zero variance will be excluded from the model. In other words, when *P*(*i*, 1) is greater than 0.90, the SNP effects will be set as zero. Previous investigation showed that 4000 MCMC iterations were required by HyB_BR for both 600 K SNP panel and imputed sequence data to maximize accuracy of genomic prediction across all the traits [16].

To compare the computational cost between BayesR and HyB_BR and how this changed with an increasing number of individuals in the reference set, we divided the data (Table 1) into three different referent sets (Ref1, Ref2, and Ref3) (with the number of sequence variants held constant). Ref1 had Holstein bulls only with 3049 bulls; Ref2 included Holstein bulls and cows with 12,527 animals; Ref3 had all the data (16,214 animals).

In all three reference sets, the speed advantage of HyB_BR compared with BayesR was investigated. Then the accuracy of genomic prediction from BayesR, HyB_BR and GBLUP was compared in the full data (including the sequence variants).

In addition, the precision of mapping QTL from the three methods was compared.

## Results

### Computational time comparison between GBLUP, BayesR and HyB_BR

**Fig. 2:**
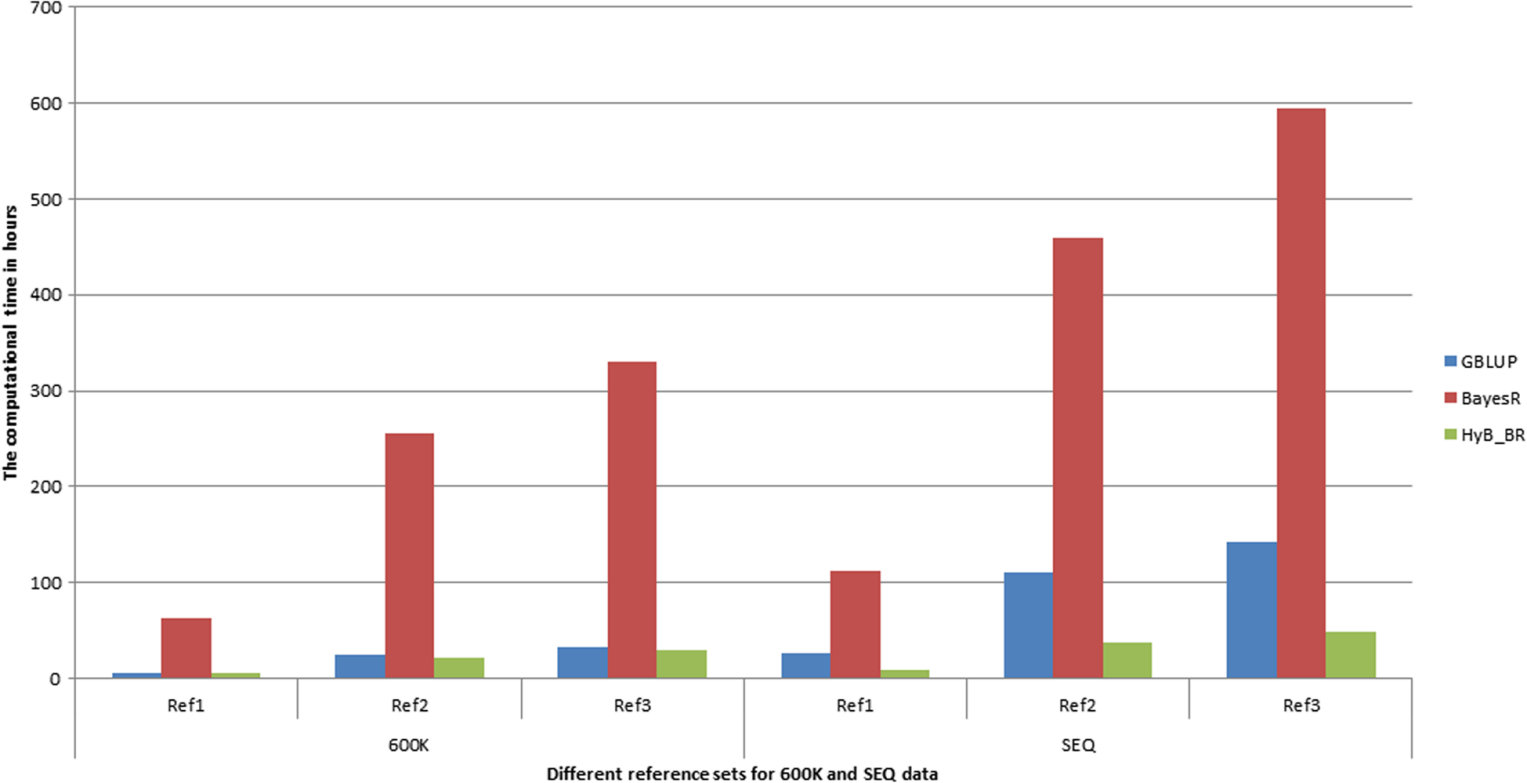
The computational time comparison between GBLUP, BayesR and HyB_BR on 600 K and SEQ data. Three reference sets (Ref1, Ref2 and Ref3) with the same number of variants (600 K or SEQ) are used here. Ref1 has Holstein bulls data with 3049 animals; Ref2 has Holstein bulls and cows data with 12,527 animals; Ref3 has Holstein and Jersey bulls and cows with 16,214 individuals

For both 600 K and SEQ data sets, HyB_BR was more than 10 times faster than BayesR, Fig. 2. As the size of the data set increased (from Ref 1 to Ref3 or from 600 K to SEQ data), the computational time required for HyB_BR could be reduced by a greater and greater margin relative to BayesR. On 600 K data, HyB_BR had a similar compute time to GBLUP. For the SEQ data, HyB_BR was up to four fold faster than GBLUP.

These timings were recorded on a server with Intel E5–2680 2.7GHz processors and 384GB of 1333 MHz RAM.

### Accuracy of genomic prediction for GBLUP, BayesR, and hybrid with sequence data

#### Prediction accuracy for milk production traits and fertility

For the milk production and fertility traits, the combined Holstein and Jersey reference sets were used to predict three validation sets including Holstein bulls (Table 3), Jersey bulls (Table 3), and Australian Red bulls & cows (Table 4).

**Table 3:** The multi-breed prediction accuracy and bias of GBLUP, BayesR, and HyB_BR on SEQ data related to Fat Yield, Milk Yield, Protein Yield, Fat%, Protein% and Fertility

		Holstein and Jersey reference to predict Holstein validation											
		Fat Yield		Milk Yield		Protein Yield		Fat%		Protein%		Fertility	
		Acc.	Bias	Acc.	Bias	Acc.	Bias	Acc.	Bias	Acc.	Bias	Acc.	Bias
GBLUP	+Poly^a^	0.64	1.07	0.66	0.92	0.63	0.95	0.76	0.95	0.83	0.98	0.42	1.70
	-Poly^b^	0.62	1.32	0.60	0.83	0.58	1.15	0.75	1.01	0.81	1.09	0.42	1.70
BayesR	+Poly^a^	0.65	1.27	0.69	0.91	0.68	1.04	0.81	1.01	0.83	0.99	0.42	1.32
	-Poly^b^	0.63	1.17	0.67	0.85	0.65	0.91	0.80	1.01	0.82	0.96	0.42	1.32
HyB_BR	+Poly^a^	0.66	1.04	0.69	0.89	0.68	0.96	0.81	0.99	0.83	0.96	0.42	1.32
	-Poly^b^	0.63	0.96	0.69	0.89	0.66	0.88	0.81	0.99	0.81	0.94	0.42	1.32
		Holstein and Jersey reference to predict Jersey validation											
		Fat Yield		Milk Yield		Protein Yield		Fat%		Protein%		Fertility	
		Acc.	Bias	Acc.	Bias	Acc.	Bias	Acc.	Bias	Acc.	Bias	Acc.	Bias
GBLUP	+Poly^a^	0.54	0.76	0.65	0.88	0.69	0.94	0.67	0.86	0.77	0.94	0.23	1.13
	-Poly^b^	0.52	0.93	0.65	1.03	0.68	1.24	0.66	0.93	0.75	1.02	0.23	1.13
BayesR	+Poly^a^	0.57	0.88	0.70	0.96	0.72	1.22	0.77	0.97	0.77	0.89	0.23	1.03
	-Poly^b^	0.52	0.73	0.68	0.87	0.67	1.02	0.76	0.95	0.77	0.87	0.23	1.02
HyB_BR	+Poly^a^	0.58	0.87	0.69	0.95	0.73	0.91	0.77	0.93	0.79	0.87	0.23	0.97
	-Poly^b^	0.57	0.74	0.69	0.85	0.73	0.91	0.76	0.93	0.78	0.85	0.23	0.97

The bulls and cows from two breeds of Holstein and Jersey are used as the reference set to predict Holstein bulls and Jersey bulls separately. ^a^The prediction accuracy when adding the polygenic term in the model; while^b^is the prediction accuracy when leaving out the polygenic term from the model

**Table 4:** The across breed prediction accuracy of GBLUP, BayesR, and HyB_BR on SEQ data related to Fat Yield, Milk Yield, Protein Yield, Fat%, Protein% and Fertility

	Across breeds prediction on Australian red bulls											
	Fat Yield		Milk Yield		Protein Yield		Fat%		Protein%		Fertility	
	Acc.	Bias	Acc.	Bias	Acc.	Bias	Acc.	Bias	Acc.	Bias	Acc.	Bias
GBLUP	0.13	0.58	0.21	0.59	0.15	0.71	0.39	0.61	0.50	1.32	0.22	0.96
BayesR	0.35	1.31	0.22	0.77	0.24	0.92	0.40	0.61	0.53	0.86	0.27	0.97
HyB_BR	0.28	0.74	0.36	0.70	0.26	0.74	0.47	0.66	0.53	0.88	0.27	0.95
	Across breeds prediction on Australian red cows											
	Fat Yield		Milk Yield		Protein Yield		Fat%		Protein%		Fertility	
	Acc.	Bias	Acc.	Bias	Acc.	Bias	Acc.	Bias	Acc.	Bias	Acc.	Bias
GBLUP	0.15	0.77	0.11	0.37	0.12	0.57	0.31	0.92	0.34	1.09	0.07	0.61
BayesR	0.28	1.02	0.22	0.55	0.16	0.60	0.37	0.94	0.34	0.93	0.07	0.52
HyB_BR	0.25	0.88	0.23	0.54	0.16	0.59	0.37	0.91	0.34	0.91	0.07	0.57

The bulls and cows from two breeds of Holstein and Jersey are used as the reference set to predict Australian red bulls and cows

When predicting the Holstein validation bull data, BayesR and HyB_BR performed equally well. Compared with GBLUP, BayesR and HyB_BR had a small but consistent accuracy improvement for the milk production traits except protein%. For fat% trait, BayesR and HyB_BR gave a 5% improvement in accuracy compared with GBLUP. However, for protein% and fertility there was no difference between the methods. With the Jersey validation set, the accuracy superiority of HyB_BR and BayesR over GBLUP was greater; for example for fat percent, BayesR and HyB_BR gave a 10% higher accuracy than GBLUP. HyB_BR and BayesR also gave regression coefficients (DTD on GEBV) closer to one than GBLUP for most traits.

In addition, when incorporating the polygenic effects into the prediction model, a small but consistent accuracy improvement was observed for milk production traits, Table 3. However, for fertility, including the polygenic effects did not affect the prediction accuracy at all.

When predicting Australian red bulls and cows using the combined Holstein and Jersey reference set (across breed prediction), both HyB_BR and BayesR had a considerable accuracy advantage (up to 12% increase) over GBLUP for all the traits (Table 4). Compared with BayesR, HyB_BR performed equally, or better, in terms of accuracy for all traits except fat yield.

#### Accuracy of genomic prediction for heat tolerance

The accuracy of genomic prediction for the heat tolerance traits was similar for GBLUP, BayesR, and HyB_BR, Table 5. There were two exceptions when predicting the validation set of Jersey bulls: 1) for the fat yield trait associated heat tolerance, there was a 6% accuracy reduction for BayesR and HyB_BR in comparison with GBLUP; 2) For milk yield, a 9% increase in accuracy from BayesR and HyB_BR over that of GBLUP was observed. Given the small size of the validation populations, these differences were not statistically significantly different. HyB_BR and BayesR did give regression coefficients closer to one compared with GBLUP for all the traits.

**Table 5:** The multi-breed prediction accuracy and bias of GBLUP, BayesR, and HyB_BR on SEQ data related to traits affected by heat tolerance

	Holstein and Jersey reference Prediction on Holstein bulls					
	Fat		Milk		Protein	
	Acc.	Bias	Acc.	Bias	Acc.	Bias
GBLUP	0.35	1.47	0.24	0.84	0.32	1.24
BayesR	0.35	1.05	0.29	0.88	0.33	0.92
HyB_BR	0.35	1.05	0.28	0.86	0.33	1.01
	Holstein and Jersey reference Prediction on Jersey bulls					
	Fat		Milk		Protein	
	Acc.	Bias	Acc.	Bias	Acc.	Bias
GBLUP	0.33	1.25	0.37	1.11	0.35	0.72
BayesR	0.27	0.89	0.46	0.89	0.35	0.76
HyB_BR	0.27	0.88	0.46	0.89	0.35	0.77

The bulls and cows from two breeds of Holstein and Jersey are used as the reference set to predict Holstein bulls and Jersey bulls separately

**Fig. 3:**
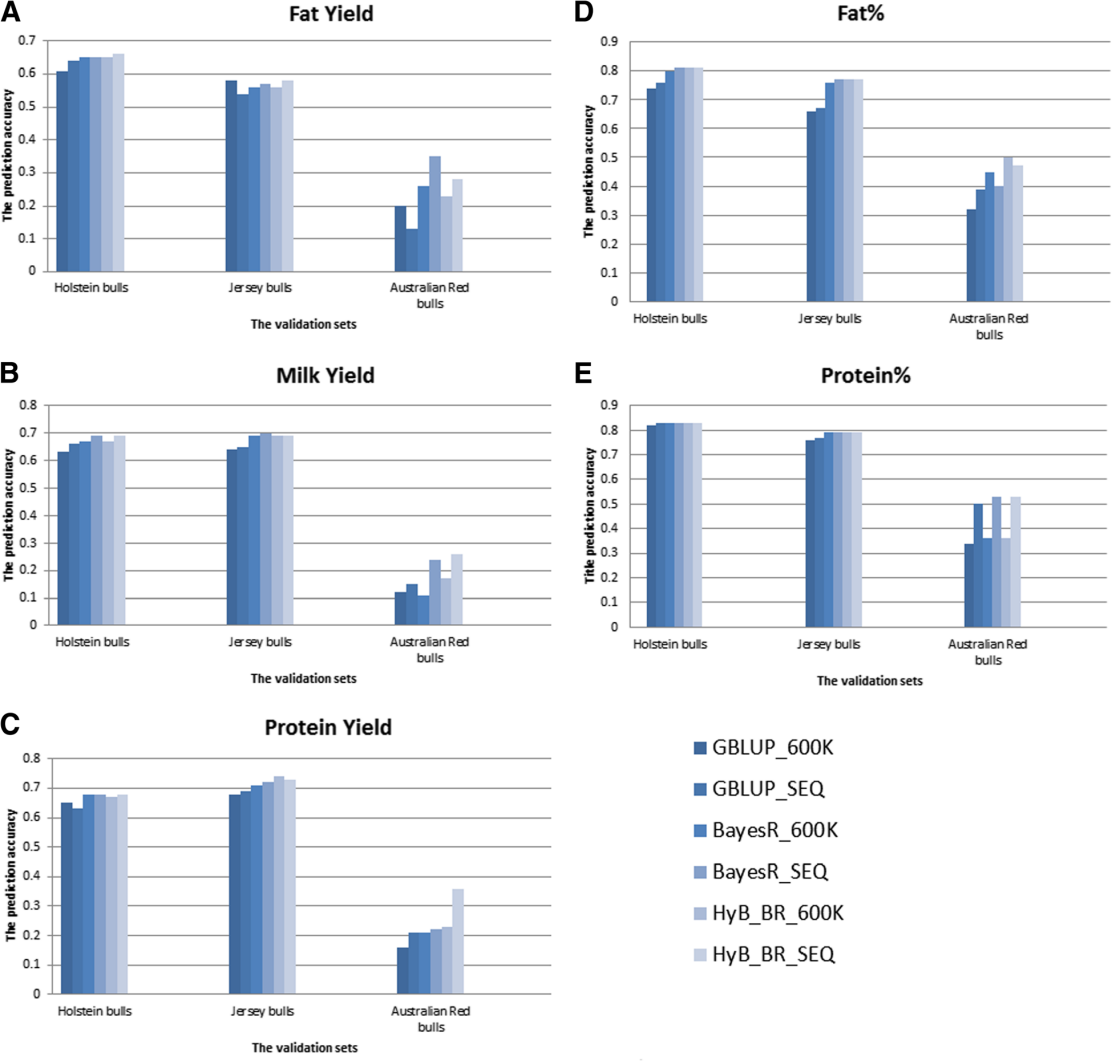
The prediction accuracy of GBLUP, BayesR, and HyB_BR on 600 K and SEQ data related to three milk production traits including Fat Yield (**a**), Milk Yield (**b**), Protein Yield (**c**), Fat Percent (**d**), and Protein Percent (**e**)

Compared with 600 K SNP panels, the impact of sequence data (SEQ) on the prediction accuracy of GBLUP, BayesR, and HyB_BR was dependent on the trait and validation population (Fig. 3). For the prediction of the validation sets of Holstein or Jersey bulls (which were closely related to the reference set), only a very small accuracy gain (1% ~ 2%) was observed from using sequence data compared to using the 600 K panel. However, when the validation set comprised of Australian Red bulls and cows, there was greater advantage of using the sequence data, provided BayesR or HyB_BR was used. For example, the accuracy using BayesR and HyB_BR with the sequence data was up to 13% higher than when the 600 K SNP panel was used. When using sequence data, GBLUP gave only a very limited increase (or even a reduction for Fat Yield trait).

### Inference of genetic architecture

To compare the genetic architecture of the traits using whole genome sequence data, the number of SNPs in each of four distributions (with the variance ${0}^{\ast }{\sigma}_g^2$, ${0.0001}^{\ast }{\sigma}_g^2$, ${0.001}^{\ast }{\sigma}_g^2$, or ${0.01}^{\ast }{\sigma}_g^2$) was investigated (Table 6). Across all the traits, BayesR and HyB_BR gave a similar proportion of SNP in the distribution with the largest variance $0.01{\sigma}_g^2$. However, there was a difference in the proportion of SNPs in each of the four distributions, in that is HyB_BR systematically estimated more variants in the distributions with non-zero variances than BayesR.

**Table 6:** The proportion of variants in each of four distributions (0, ${0.0001}^{\ast }{\sigma}_g^2$, ${0.001}^{\ast }{\sigma}_g^2$, or ${0.01}^{\ast }{\sigma}_g^2$) estimated from BayesR (termed BR) and HyB_BR (termed HB)

	Fat Yield		Milk Yield		Protein Yield		Fat%		Protein%		Fertility	
	BR (%)	HB (%)	BR (%)	HB (%)	BR (%)	HB (%)	BR (%)	HB (%)	BR (%)	HB (%)	BR (%)	HB (%)
0	99.484	99.015	99.542	99.242	99.494	99.224	99.660	99.380	99.577	99.171	99.515	99.298
$0.0001{\sigma}_g^2$	0.513	0.958	0.449	0.717	0.501	0.725	0.333	0.612	0.405	0.799	0.453	0.676
$0.001{\sigma}_g^2$	0.002	0.027	0.009	0.039	0.004	0.05	0.004	0.006	0.015	0.028	0.030	0.025
$0.01{\sigma}_g^2$	0.001	0.001	0.001	0.002	0.001	0.001	0.002	0.002	0.002	0.002	0.001	0.002

### QTL mapping

**Fig. 4:**
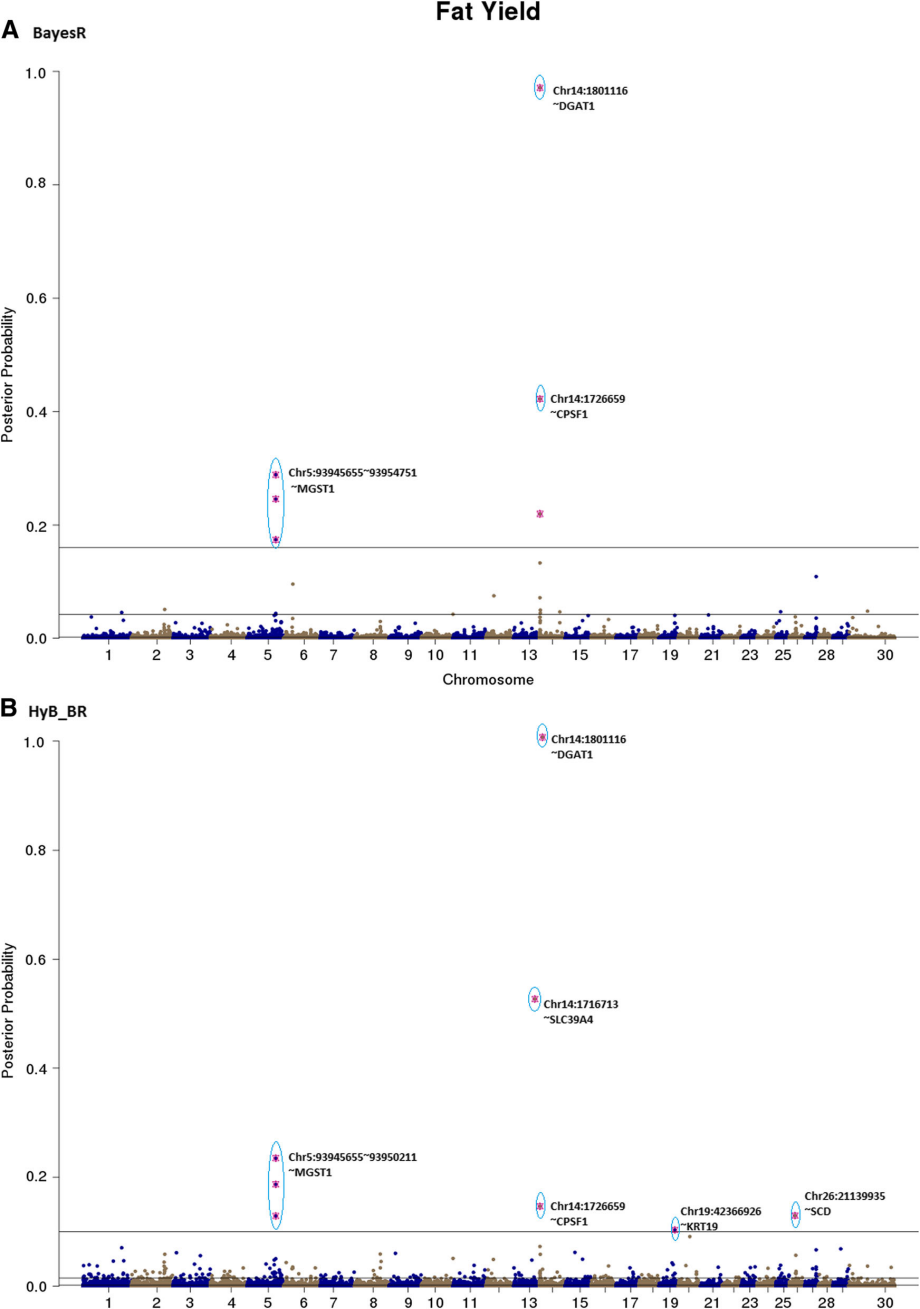
Posterior possibilities of all the variants on fat yield estimated from BayesR (**a**) and HyB_BR (**b**) according to their positions (base pairs) across the whole genome. The top SNPs with highest posterior possibilities are labelled with *blue circle*

**Fig. 5:**
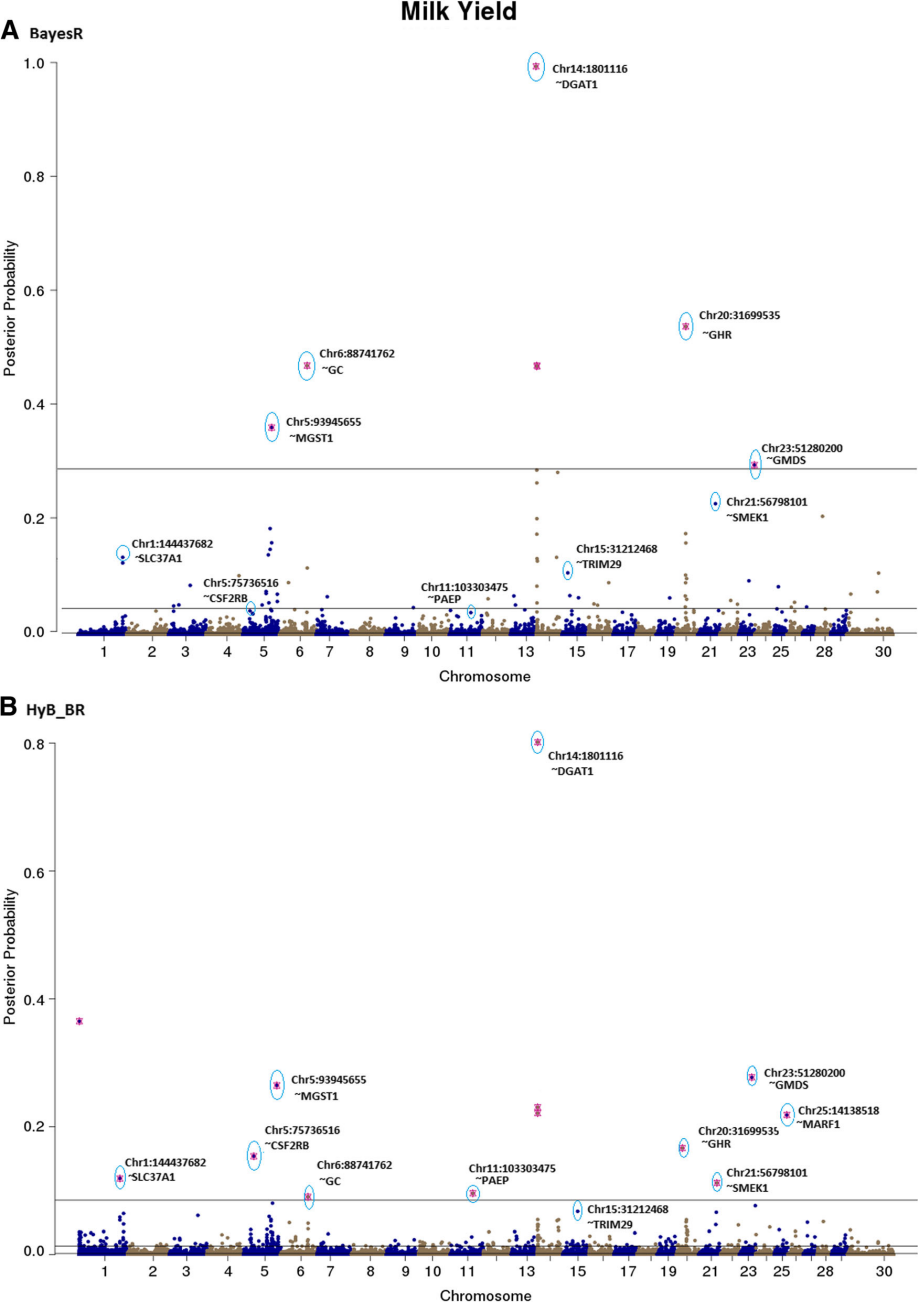
Posterior possibilities of all the variants for milk yield estimated from BayesR (**a**) and HyB_BR (**b**) according to their positions (base pairs) across the whole genome. The top SNPs with highest posterior possibilities are labelled with *blue circle*

**Fig. 6:**
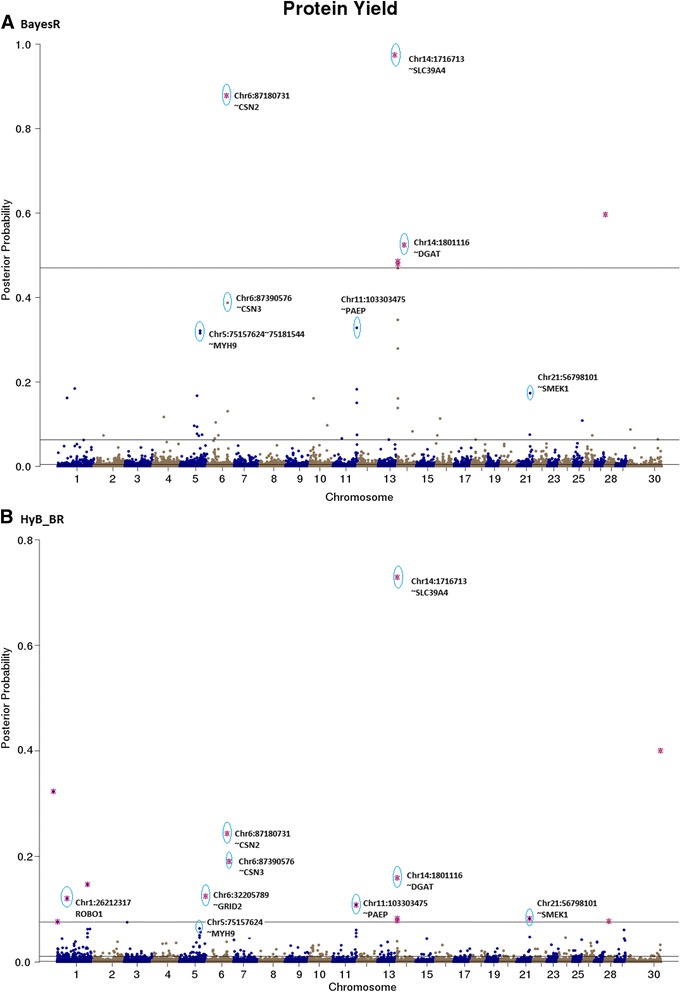
Posterior possibilities of all the variants for protein yield estimated from BayesR (**a**) and HyB_BR (**b**) according to their positions (base pairs) across the whole chromosome genome. The top SNPs with highest posterior possibilities are labelled with *blue circle*

**Fig. 7:**
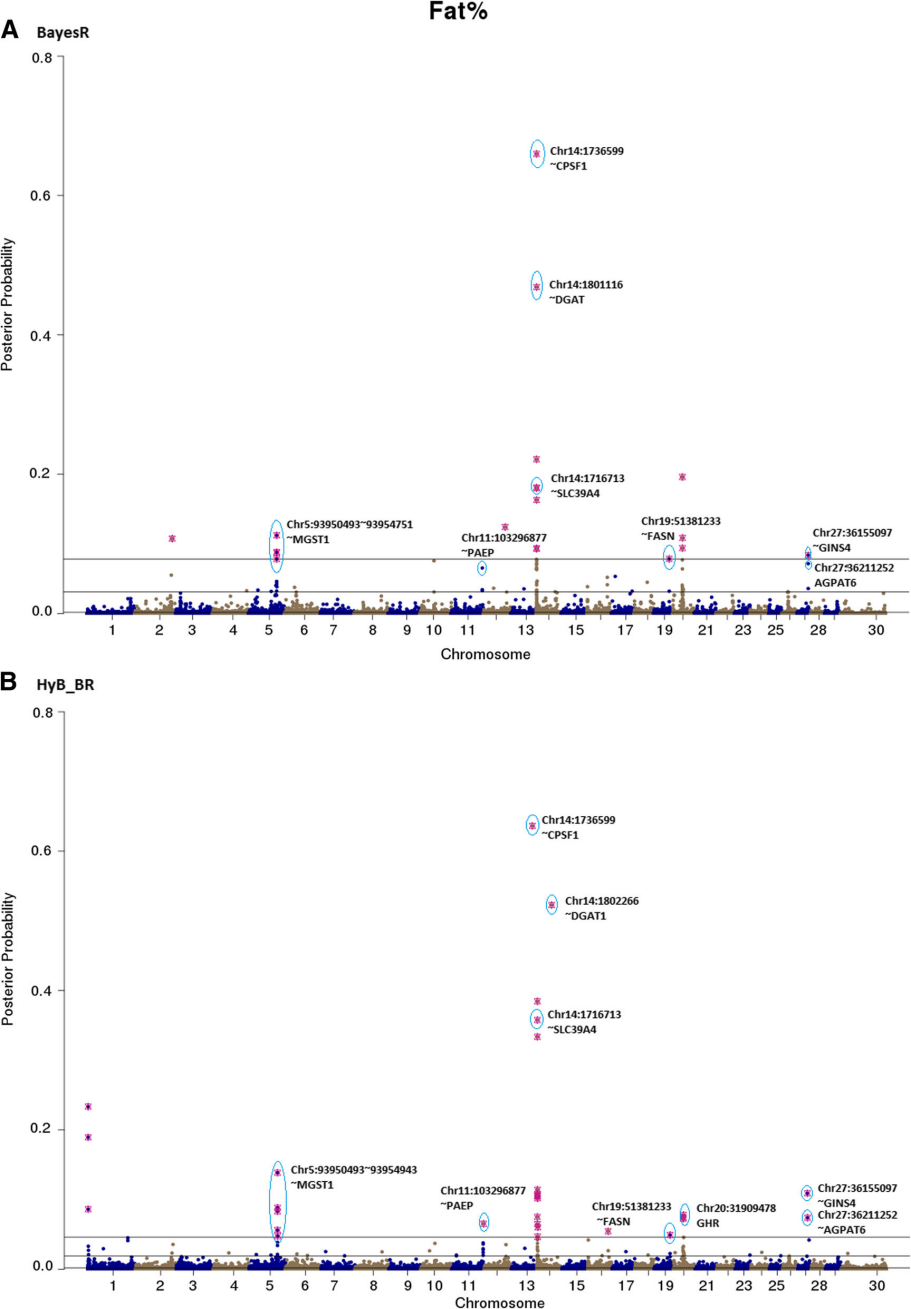
Posterior possibilities of all the variants for fat percent estimated from BayesR (**a**) and HyB_BR (**b**) according to their positions (base pairs) across the whole genome. The top SNPs with highest posterior possibilities are labelled with *blue circle*

**Fig. 8:**
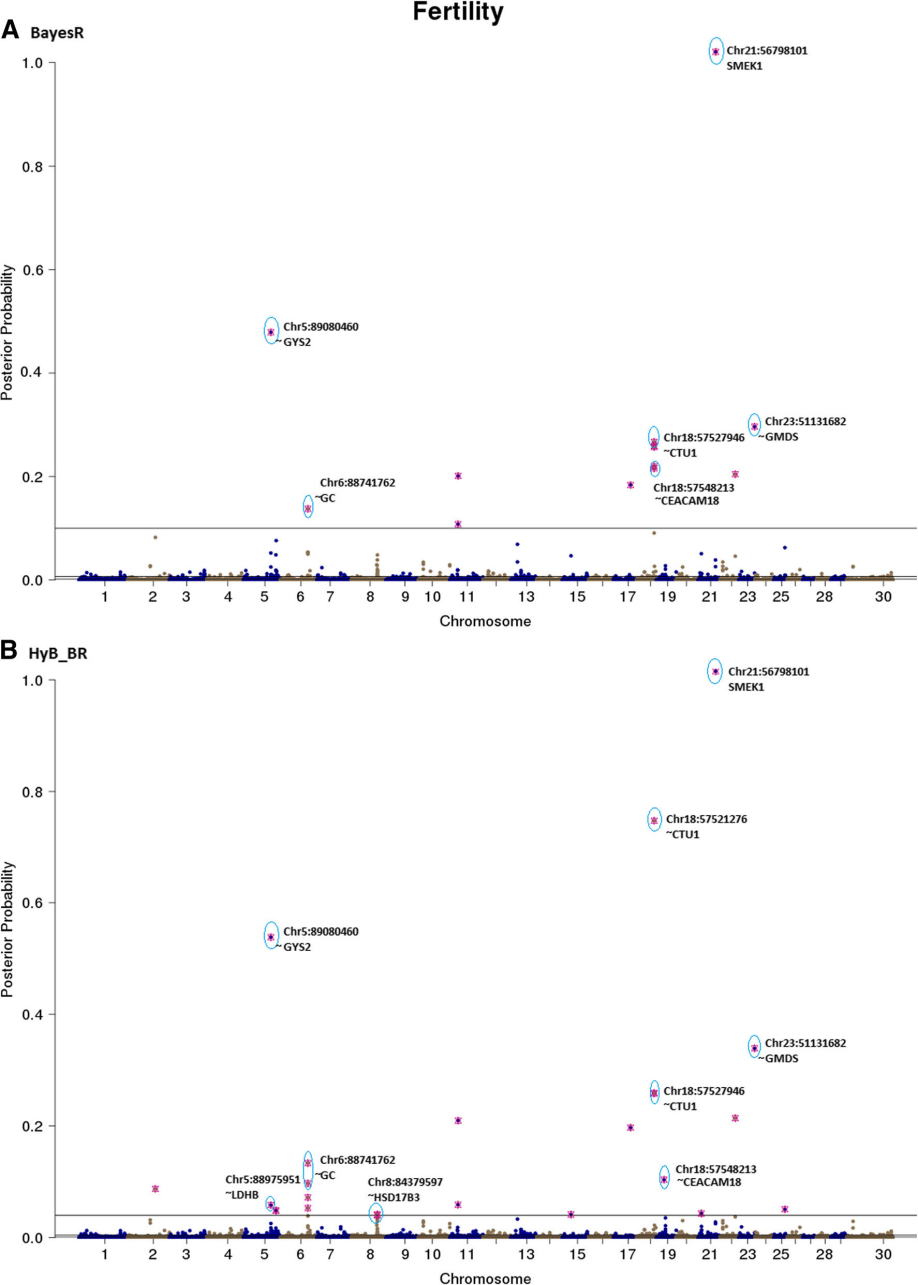
Posterior possibilities of all the variants on fertility estimated from BayesR (**a**) and HyB_BR (**b**) according to their positions (base pairs) across the whole genome. The top SNPs with highest posterior possibilities are labelled with *blue circle*

**Fig. 9:**
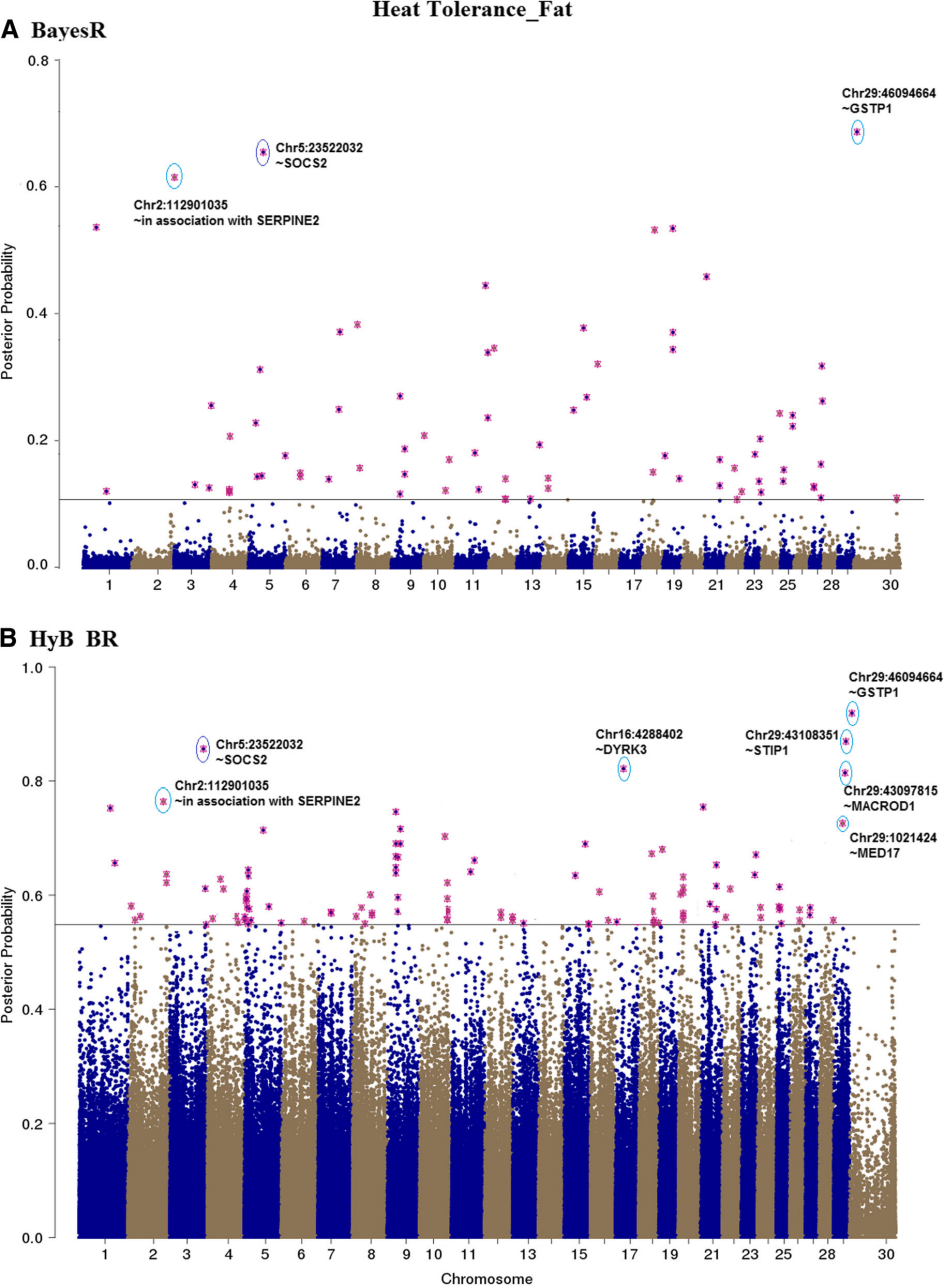
Mapping posterior probabilities of all the variants estimated from BayesR (**a**) and HyB_BR (**b**) according to their positions (base pairs) across the whole chromosome related to Fat yield affected by heat tolerance. The top SNPs with highest posterior possibilities are labelled with *blue circle*

**Fig. 10:**
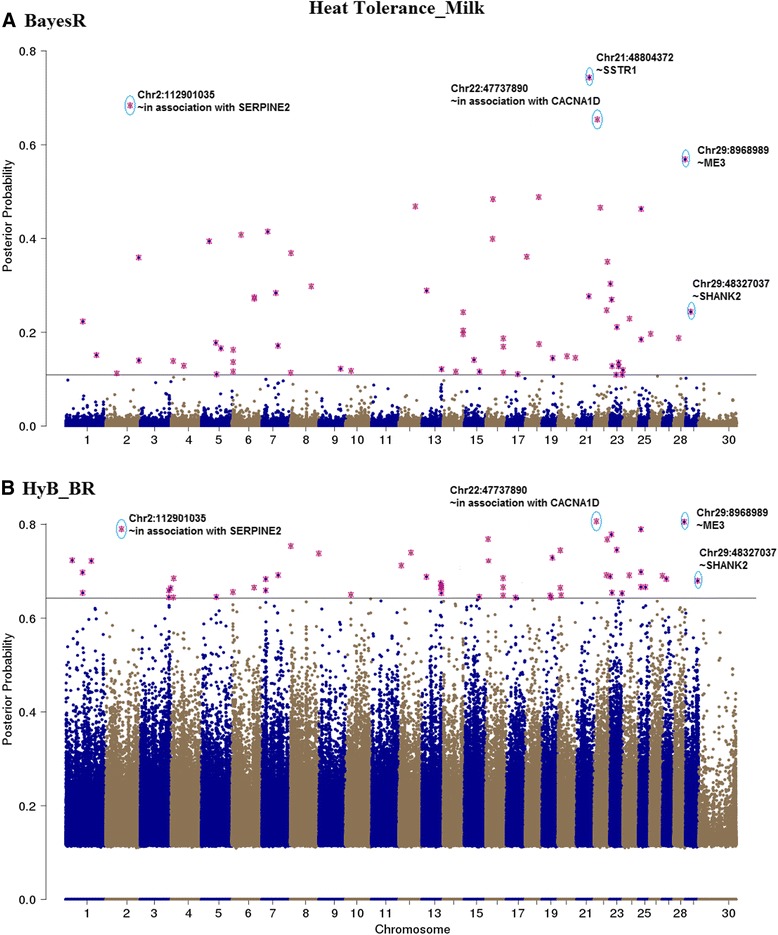
Mapping the posterior probabilities of all the variants estimated from BayesR (**a**) and HyB_BR (**b**) according to their positions (base pairs) across the whole chromosome related to Milk yield affected by heat tolerance. The top SNPs with highest posterior possibilities are labelled with *blue circle*

**Fig. 11:**
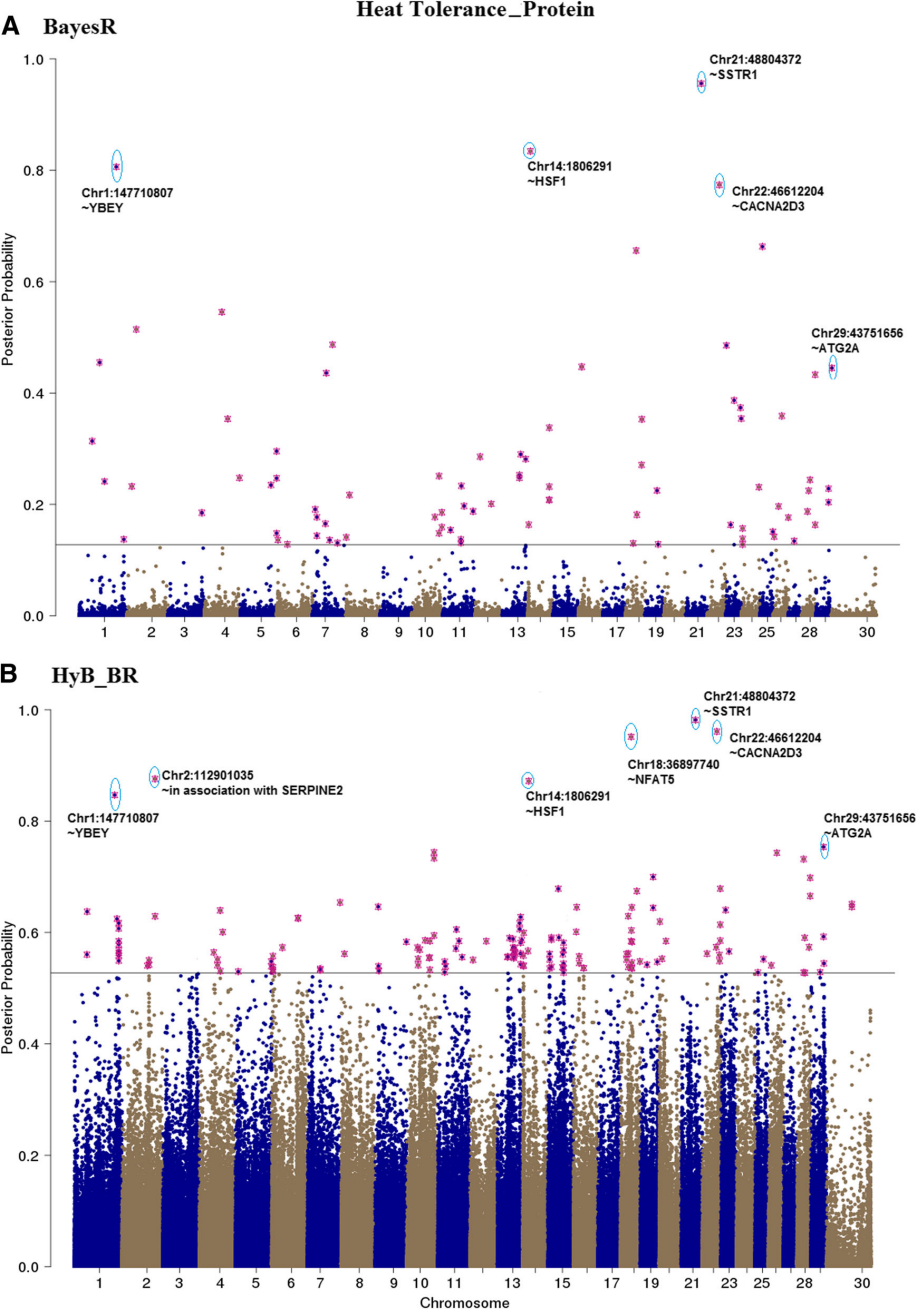
Mapping the posterior probabilities of all the variants estimated from BayesR (**a**) and HyB_BR (**b**) according to their positions (base pairs) across the whole chromosome related to protein yield affected by heat tolerance. The top SNPs with highest posterior possibilities are labelled with *blue circle*

For all the traits, estimated posterior possibilities from BayesR and HyB_BR were plotted across the whole genome locations of SNPs, Figs. 4, 5, 6, 7, 8, 9, 10 and 11. According to the posterior possibilities, the thresholds (the grey horizon lines in the figures; the probabilities above which there are the same number of SNPs as in the distribution with largest variance ${0.01}^{\ast }{\sigma}_g^2$) were set to highlight the top SNPs. Top variants with the highest posterior probability of being in the distribution with the largest variance from BayesR and HyB_BR were investigated.

#### QTL mapping for milk production traits

The top variants detected by both BayesR and HyB_BR (Table 7) were in, or close to, many previously described genes involved with milk production. For example, in Table 7, some well-known mutations impacting milk synthesis included DGAT1 [29–31], FASN [32], SCD [33], PAEP [34], AGPAT6 [35, 36], and CNS2/3 [5]. Notably, for the trait Fat% (Fig. 7), HyB_BR was able to find the real causal mutation in the DGAT1 gene, located at 1802266 bp of Chromosome 14, which has been reported by Grisart et al., 2004 [29]. In addition, HyB_BR could detect some novel potential causal mutations including in the genes GC (encoding the vitamin D binding protein, affecting milk yield), SMEK1 (regulating the Insulin/IGF pathway, indirectly impacting milk production and fertility) and MYH9 (myosin, heavy chain 9, non-muscle; impacting protein yield [5, 37, 38].

**Table 7:**
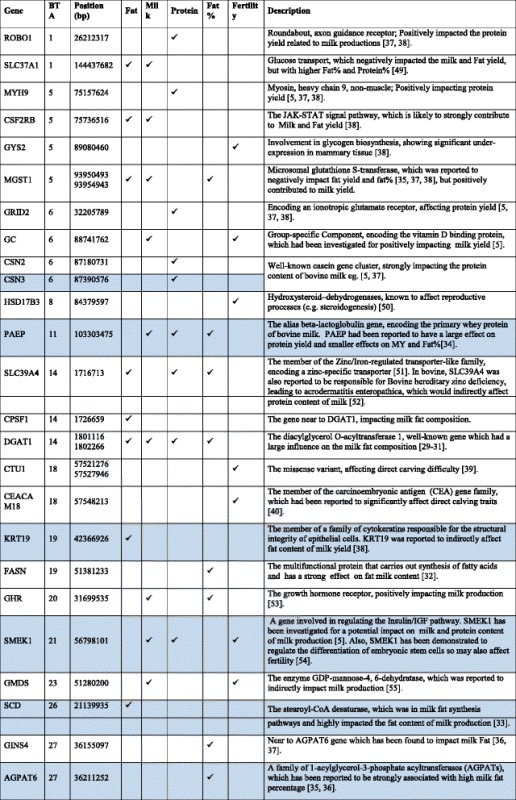
Known genes (impacting milk production traits and fertility) identified by HyB_BR using the variants with the largest variances ${0.01}^{\ast }{\sigma}_g^2$ [49–55]

The blue bar highlights the genes that were not detected by BayesR in the proportion with the largest variances

#### QTL mapping for fertility

For fertility, a putative candidate gene located on Chromosome 18 including (around genes CTU1 and CEACAM18) was detected by BayesR and HyB_BR. These genes haveb previously been reported to be associated with calving traits [39, 40].

#### QTL mapping for heat tolerance traits

As there is a significant unfavourable correlation between milk production and heat tolerance, at least for the traits we have used for heat tolerance (decline in milk production with increasing heat stress) [21], mutations that affect milk production are also likely to affect heat tolerance. To avoid detecting just QTL with large effects on milk production, QTL mapping for heat tolerance traits was performed fitting fixed effects of the mutations in DGAT1, ROBO1, PAEP, and MGST1 (the mutations with largest effects on milk production, to ensure these mutations were not picked up again in the heat tolerance mapping) in the BayesR and HyB_BR models. The posterior possibilities of all the variants estimated by HyB_BR and BayesR were plotted across the whole genome sequence in Figs. 9, 10, and 11. Compared with BayesR, HyB_BR systematically detected more SNPs with small effects (${0.001}^{\ast }{\sigma}_g^2$) while identifying fewer SNP with zero effects.

In total, we found fourteen novel variants (Table 8) in our study which have previously been associated with heat tolerance in humans or other species. YBEY [41, 42], located at BTA1 with the position 147,710,807 bp, has been reported to be important in the response of infection of *Escherichia coli* of human or other animals under heat-shock response. Variants in SERPINE2 and CACNA1D (close to the variants detected in our study, BTA2:112,901,035 and BTA22:47,737,890 respectively) have been reported to impact the sweating rate and respiration rate of dairy cattle [43]. DYRK3 (The dual specificity tyrosine-phosphorylation-regulated kinase 3), has been reported to affect respiration rate (breaths per minute) in dairy cattle [43]. HSF1, heat shock factor protein 1, coordinates stress-induced transcription in Human [44]. One single nucleotide polymorphism (SNP) in the 3′-untranslated region (g.4693G > T) of HSF1 has been reported to be in association with thermo tolerance in Chinese Holstein cattle [45]. STIP1, stress inducible protein 1, has been reported to be homologous to hsc70/hsp90 in human [46]. In mice, STIP1 could play a key role on in the ability of germ cells to survive in stress conditions including high temperatures [47]. Further investigation of the effect of these genes on heat tolerance is required.

**Table 8:** Known genes interacting with heat stress

Gene	BTA	Position	Traits			Description
			Fat	Milk	Protein	
YBEY	1	147,710,807			✓	The translation-associated heat shock genes, playing key roles in the heat-shock response of *E. coli* under heat shock stress [41, 42].
Unknown	2	112,901,035	✓	✓	✓	In association with the gene SERPINE2, which had been proven to impact the sweating rate of dairy cattle [43]
SOCS2	5	23,522,032	✓			Suppressor of cytokine signalling 2, might be responsible for heat stress abatement during the dry period of dairy cattle [56].
HSF1	14	1,806,291			✓	Genes involved in the bovine heat stress response [45, 57].
DYRK3	16	4,288,402	✓			The dual specificity tyrosine-phosphorylation-regulated kinase 3, impacting Respiration rate (breaths per minute) in dairy cattle [43]
NFAT5	18	36,897,740			✓	Nuclear factor of activated T cells, simulating transcription of Heat shock protein 70 [58].
SSTR1	21	48,804,372			✓	Somatostatin receptor 1, playing a role in heat stress sensing or communicating stress status between cells [59].
CACNA2D3	22	46,612,204			✓	Methylation of the Calcium Channel-Related Gene, showing impaired behavioural heat pain sensitivity in mice and human studies [60].
MED17	29	1,021,424	✓			The mediator mutant yeast, which was temperature-sensitive [61].
ME3	29	8,968,989		✓		Malic Enzyme 3, conferring heat-stable resistance to root-knot nematodes in plants [62].
MACROD1	29	43,097,815	✓			Heat shock protein 90 kDa alpha (cytosolic), class A member 1, which might be in association with PAR (had been proved to function heat shock response) [63, 64].
STIP1	29	43,108,351	✓		✓	Stress inducible protein 1, was homologous to the human heat shock cognate protein 70 (hsc70)/heat shock protein 90 (hsp90) [47].
GSTP1	29	46,094,664	✓			Glutathione S-transferase Pi, which was reported to play a positive role under heat stress in controlling cellular toxicants and to alleviate the destructive effect on cattle [65].
ATG2A	29	43,751,656			✓	Autophagy Related 2 Homolog A, which had been referred to as the Heat Stress-repressed target genes by Niskanen et al., 2015 [66].

All the listed genes are identified by HyB_BR using the variants with the largest variances ${0.01}^{\ast }{\sigma}_g^2$

## Discussion

In this paper, we have demonstrated that HyB_BR [16] could be efficiently implemented for simultaneous prediction of genomic estimated breeding values, inference of genetic architecture, and potential causal mutation discovery using whole-genome sequence data. As mentioned by Wang et al. (2016), HyB_BR was developed to overcome two challenges:

1. Long compute times are the main limitation of traditional MCMC Bayesian models applied to whole genome sequence data with very large data size. Therefore, an Expectation-Maximisation scheme was introduced to reduce number of iterations of MCMC.
2. Fast schemes (mainly including Iterative Conditional Expectation, and Expectation-Maximisation algorithms) implemented for Bayesian models have tended to reduce the accuracy compared with MCMC.

HyB_BR implements an EM algorithm to quickly converge for estimates of SNP effects and other parameters, followed by a limited number of MCMC iterations to optimise the posterior estimation for SNP effects. When applied to whole genome sequence data, our results indicated HyB_BR had similar accuracy of genomic prediction and precision of QTL mapping to BayesR implemented with full MCMC, but with 10 fold less computational time required. Furthermore, compared with the prediction accuracy on 600 K SNP panels, we have demonstrated that using sequence data improved the accuracy of genomic prediction for some of the traits, and particularly in multi-breed evaluations, if a breed was not included in the reference population.

The key improvement for computational efficiency was that HyB_BR reduced the iteration times. BayesR required a huge number of MCMC iterations, which was dependent on the size of the data. For example, on the whole genome sequence data with 16,214 animals and almost 1 million variants, 40,000 iterations with first 20,000 as burn-in were required. For each MCMC iteration, the basis operation times were *O*(*mn* ^2^). In comparison with BayesR, HyB_BR has the same number of basic operations. But after the EM converges (with very small number of iterations as demonstrated by Wang et al. (2015) [27]), HyB_BR implemented MCMC iterations with speed-up schemes, which could reduce the iteration number to 4000 iterations. The results from Fig. 2 provided the evidence that HyB_BR was up to 10 times faster than BayesR in the whole genome sequence data set.

In addition to the computational time, the prediction accuracy of HyB_BR for multi-breed prediction and across-breed prediction was very similar to BayesR for a range of traits with various genetic architectures, shown in Tables 3, 4 and 5. The accuracy advantage of HyB_BR and BayesR over GBLUP for across-breed prediction demonstrated the benefit of the non-linear Bayesian models. Also, the increase in accuracy using whole genome sequence data for across-breed prediction in comparison with using 600 K data, confirmed the results from [5].

For the genetic architecture identification of milk production traits, there was one notable difference between BayesR and HyB_BR: In comparison with BayesR, HyB_BR does not shrink variants with small effects (${0.001}^{\ast }{\sigma}_g^2$) as strongly, the same is true for very small effects (${0.0001}^{\ast }{\sigma}_g^2$), (Table 6). The same is true for the identification of causal mutations for heat tolerance, Figs. 9, 10 and 11. One explanation is that EM steps do not have enough power to shrink SNPs with small effects [27], which limits the following MCMC steps.

For the heat tolerance traits, there is relatively little literature reporting QTL for heat tolerance in cattle. Only one of the additive genetic variants (located at Chromosome 29 with the position 48,329,079 base pairs; close to FGF4) [48], later suggested to be SHANK2 by [43] has previously been reported. In Table 7, the gene SHANK2 was detected but not in the list of top causal mutations. However, both BayesR and HyB_BR did pick up mutations in or close to seven genes (e.g. YEBY, HSF1, MED17, ME3, STIP1, SERPINE2 and CACNA1D), which have been reported by previous studies to be involved in response to heat stress events in cattle (e.g. [45]), human, mice, or other species. In addition, HyB_BR also detected two other unknown variants. All these variants required the further investigation in regards to their function interacting between milk productions and heat tolerance.

The computational advantage of HyB_BR makes it attractive for implementation of genomic prediction in many applications. However, there are still two limitations: 1) the speed-up scheme of HyB_BR defines the fixed threshold for different traits and various densities of genomic data, which could hinder its flexibility for practical applications; 2) when the size of the data increases dramatically to 30 million variants on millions of animals, which is possible in the near future, HyB_BR is still not computationally efficient enough. Therefore, a flexible and more efficient speed-up scheme will play an important role to further improve the computational performance of HyB_BR.

## Conclusion

A hybrid scheme of Expectation-Maximisation algorithm and MCMC sampling was implemented on whole-genome sequence data for simultaneous genomic prediction, inference of genetic architecture inference and causal mutation identification. The accuracy of HyB_BR for multi-breed and across breed prediction for all traits was very similar to the results from BayesR (implemented with full MCMC) while requiring only 1/10 of the total running time of BayesR. HyB_BR could identify some well-known mutations (e.g. DGAT1) with the highest posterior probability, which demonstrated the value of the method for QTL mapping of complex traits. The advantage of using sequence data and HyB_BR was greatest for multi-breed and across breed predictions.
